# Family and Aging in Cross-National Contexts

**DOI:** 10.1093/geroni/igab046.1518

**Published:** 2021-12-17

**Authors:** Jennifer Ailshire, Jennifer Ailshire

## Abstract

The global aging of the population, combined with shifts in the structure and composition of families, has led to increased attention to the role of family and social relationships in the aging experience. The importance of family in determining healthy aging, however, may largely depend on the social, political, and economic context in which individuals are embedded. Cross-national investigations offer a unique opportunity to understand how family relationships and family caregiving influence health and well-being among older adults by comparing family dynamics across different sociocultural contexts. The HRS-family of surveys, which have been harmonized within the Gateway to Global Aging, provides remarkable opportunities for cross-national comparative analysis. The papers in this session use harmonized data from the Gateway to compare the influence of family on health across different social dimensions in multiple countries from around the world, including examinations of: 1) the impact of grandparenting on health of older adults in Europe and China; 2) psychological well-being among European older adults whose partners receive formal care; 3) the influence of parent-child relationships on health and well-being of older adults in China and the U.S.; and 4) how loneliness among older adults is patterned according to their living arrangements and relationship quality. The discussion will highlight the promises and challenges of cross-national research on families and aging and how harmonized aging data facilitates international comparisons.

